# The Role of Fecal Microbiota Transplantation in the Treatment of Acute Graft-versus-Host Disease

**DOI:** 10.3390/biomedicines10040837

**Published:** 2022-04-01

**Authors:** Jarosław Biliński, Marcin Jasiński, Grzegorz W. Basak

**Affiliations:** 1 Department of Hematology, Transplantation and Internal Medicine, Medical University of Warsaw, 02-091 Warsaw, Poland; marcin.jasinski@wum.edu.pl (M.J.); grzegorz.basak@wum.edu.pl (G.W.B.); 2 Human Biome Institute, 80-137 Gdansk, Poland; 3 Doctoral School, Medical University of Warsaw, 02-091 Warsaw, Poland

**Keywords:** gut microbiota, graft-versus-host disease, fecal microbiota transplantation, gut-immune cells cross-talk, gastrointestinal acute GvHD

## Abstract

The number of allogeneic hematopoietic stem cell transplantations conducted worldwide is constantly rising. Together with that, the absolute number of complications after the procedure is increasing, with graft-versus-host disease (GvHD) being one of the most common. The standard treatment is steroid administration, but only 40–60% of patients will respond to the therapy and some others will be steroid-dependent. There is still no consensus regarding the best second-line option, but fecal microbiota transplantation (FMT) has shown encouraging preliminary and first clinically relevant results in recent years and seems to offer great hope for patients. The reason for treatment of steroid-resistant acute GvHD using this method derives from studies showing the significant immunomodulatory role played by the intestinal microbiota in the pathogenesis of GvHD. Depletion of commensal microbes is accountable for aggravation of the disease and is associated with decreased overall survival. In this review, we present the pathogenesis of GvHD, with special focus on the special role of the gut microbiota and its crosstalk with immune cells. Moreover, we show the results of studies and case reports to date regarding the use of FMT in the treatment of steroid-resistant acute GvHD.

## 1. Introduction

**Figure 1:**
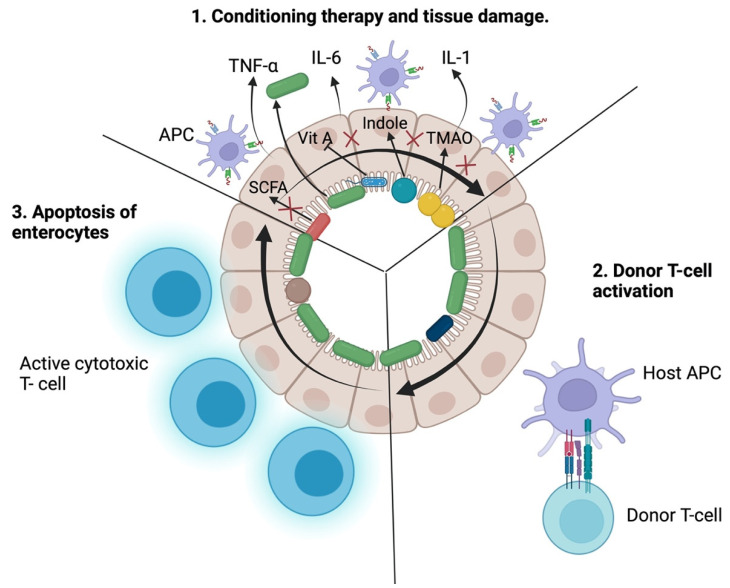
The role of the gut microbiota in the pathogenesis of graft-versus-host disease. During the first phase of GvHD, the conditioning therapy is accountable for tissue damage, and rapidly proliferating intestinal cells are particularly prone to the therapy. The damaged cells released TNFα, IL-6, and IL-1, which are known as “danger signals” that trigger the influx of APC. SCFA (short-chain fatty acids)-producing bacteria are frequently eliminated during the time of alloHCT because of the antibiotics used. Therefore, the lack of SCFA needed for maintaining intestinal integrity and inhibiting apoptosis of intestinal wall cells leads to the aggravation of GvHD. Lack of intestinal integrity results in the transfer of bacteria to the bloodstream, which can cause sepsis. Moreover, the indole that is produced by commensal microbiota is also known to play a role in maintaining intestinal integrity. TMAO (choline-derived trimethylamine N-oxide) has been shown in the mouse model to promote the differentiation of Th17 cells, which facilitates GvHD. Additionally, the level of vitamin A seems to be negatively correlated with the severity of alloHCT, and some gut microbes are known for preventing retinoid metabolism. The gut microbiota of patients with aGvHD is of poor diversity. During the second stage, the host APC presents antigens to the donor T cells, stimulating robust annihilation of the enterocytes in phase 3, which in turn leads to further aggravation of the disease.

Acute graft-versus-host disease (aGvHD) is a common life-threatening complication of allogeneic hematopoietic stem cell transplantation (alloHCT), which occurs in 25 to 50% of alloHCT recipients and is known to be the second most common cause of death (after relapse of the underlying disease) in this group of patients [1,2]. Despite its frequency having decreased over time in matched related and unrelated donor transplantations, the absolute number of patients experiencing aGvHD has increased due to the growing number of alloHCT performed worldwide [3]. The gastrointestinal manifestation of aGvHD is also increasing [4]. Graft-versus-host disease occurs when immunocompetent T cells in the graft recognize the recipient as foreign tissue and initiate the immune response (Figure 1). The main symptoms appear in the skin (maculopapular rash), liver (cholestasis due to small bile duct damage), and gastrointestinal tract (watery or bloody diarrhea, nausea, vomiting, and crampy abdominal pain). Diagnosis may be sometimes difficult because of overlapping symptoms relating to toxicities of conditioning and concomitant infections. The clinical diagnosis can be made with typical symptoms, laboratory tests, and tissue biopsy, which should be analyzed with caution because of the relatively common false-negative and false-positive results [5,6,7]. Risk factors for aGvHD are the extent of HLA disparity, increased age of both the donor and recipient, transplantation from an unrelated donor, transplantation from a female donor to a male recipient, grafts received from peripheral blood, and intensity of the conditioning regimen [8,9,10]. Prevention of GvHD includes administration of antithymocyte globulin (ATG), calcineurin inhibitors (cyclosporine or tacrolimus), and methotrexate (folate antagonist) or mycophenolate mofetil [11,12]. Acute GvHD is treated with glucocorticoids, but steroid refractoriness or dependency is relatively common and only 40–60% of patients will respond to this therapy [13]. Still, there is no consensus about the best second-line treatment, and mortality in patients not responding to steroids is high, with a six-month overall survival estimate of 50% and two-year overall survival rate of a maximum of 30% [14,15]. In 2020, for the first time, the authors of a randomized clinical trial documented that intervention with ruxolitinib resulted in statistically higher overall response rates than standard of care [16]. Among other most frequently used second-line treatments are ATG [17], post-transplant cyclophosphamide [18], etanercept [19], infliximab [20], and fecal microbiota transplantation (FMT) more recently [21].

Fecal microbiota transplantation (FMT) is probably best known for its outstanding efficacy in recurrent or refractory *Clostridioides difficile* infection (CDI), with more than 90% cure rate [17]. Together with growing evidence about the role of the gut microbiota in new entities, there are increasing attempts to reverse imbalance in the composition of gut microbiota with FMT. Our group recently presented a case series of patients treated for CDI with overlapping COVID-19, which showed only mild symptoms of the SARS-CoV-2 infection despite very high risk for a fatal course as well as a quick end to shedding of the virus in the stool [22]. Many diseases where FMT is experimentally used are characterized by a proinflammatory skewed immune response, and FMT acts as an immunoregulatory factor decreasing the vicious circle and constant production of proinflammatory cytokines. Acute GvHD is such a disease, and the first data published showed promising results of FMT as a treatment modality for steroid-refractory/dependent type of this disease [23,24,25].

## 2. Pathogenesis of Acute Graft-versus-Host Disease

Acute GvHD pathogenesis is the effect of a vicious circle that occurs when donor T lymphocytes are activated against the host tissues, which in turn release damage signals that further trigger T cells. There are three widely recognized steps in the pathogenesis of aGvHD [26,27]. During the first step (Figure 1), which is associated with tissue damage, proinflammatory cytokines, such as tumor necrosis factor-α (TNFα) or interleukin-1 (IL-1), are released. Pretransplant conditioning regimen, infections, or other inflammatory processes before the transplant are accountable for tissue damage. The released cytokines play the role of “danger signals”, which are considered to be the trigger in the pathogenesis of aGvHD [28]. Interestingly, the gastrointestinal (GI) tract plays a vital role in the cytokine release site and the whole process of aGvHD initiation [29]. Cells that are known to be particularly damaged during the conditioning therapy are Paneth and goblet cells. Paneth cells are responsible for the secretion of alpha defensins, which are antimicrobial peptides that maintain the appropriate balance in the composition of gut microbiota [30]. Loss of these cells results in intestinal dysbiosis [31]. Goblet cells secrete mucin that shields the intestinal epithelial cells (IECs) from luminal bacteria [32]. Damage of these cells causes loss of IEC integrity and permits the translocation of gut microbes and all gut antigenic load (metabolites, peptides, and proteins) into the submucosa, lymph nodes, and bloodstream. This consequently enhances the production of proinflammatory cytokines and stimulates the GvHD itself [33]. The level of damage in the GI tract is clinically of the greatest concern.

After the alloHCT procedure, step two is initiated. Donor T cells home to the lymphoid tissues within hours after transplantation. After 2–3 days, the host antigen-presenting cells (APCs) lead to donor T cell activation, proliferation, and secretion of cytokines [34]. Data suggest that nonhematopoietic APCs are predominantly engaged in this process [35]. Additionally, T cells differentiate into Th1-type, cytotoxic T cells (Tc), and Th17 lymphocytes that produce TNFα, IL-2, and IFNγ, which enhances the presentation of antigens [36]. All of these lead to cytotoxic T cells infiltration in target organs, such as the liver, GI tract, and skin [34]. The GI tract constitutes one of the barriers protecting us from the environment and is also the biggest compartment of immune-competent cells. The GI mucosa (GALT) is considered the biggest human lymphoid organ. In the second step, all APCs massively present antigens (self and nonself) to the mature donor’s T cells, thereby activating invasion and damage.

In the third step, T cells robustly proliferate in the target tissues and cause cell lysis via Fas/FasL and perforin/granzyme mechanisms. Elimination of target cells by donor cytotoxic T cells leads to a further increase in the production of proinflammatory cytokines, such as those mentioned in step one, and the vicious circle is initiated [37].

The steps in which the gut microbiota is particularly engaged is undoubtedly steps one and two. During the first phase, the GI tract is damaged, with APCs presenting in the second phase as either self (intestinal, skin, and liver epitopes) or nonself and mostly as microbial (cell wall fragments) antigens. There is mounting evidence that gut microbiota dysbiosis acts as a risk factor for GvHD. We have previously shown that pretransplant gut colonization with antibiotic-resistant bacteria, which is a marker of dysbiosis and colonization resistance loss, is a risk factor for GvHD, mostly of the GI tract [38]. Concomitantly and since then, other groups have shown that low gut bacterial diversity correlates with lower overall survival and higher incidence of GvHD [39,40]. The main cause of lower bacterial diversity is antibiotic therapy before and during alloHCT [41]. All of these facts seem logical as mucosa-associated, especially gut-associated, lymphoid tissue (MALT and GALT) contains about 70% of the lymphocyte population, and there is constant, never-ending “conversation” between immune-competent cells and the microbiota.

## 3. Crosstalk between the Intestinal Immune System and the Gut Microbiota

The wall of GI is colonized by the “two armies’’ of cells that are located on either sides of this boundary. On the one side are the gut microbes, namely bacteria, viruses, fungi, and parasites, while on the opposite side, the ‘’army’’ of immune cells is located consisting of dendritic cells (DCs), macrophages, T/B cells, and neutrophils. A boundary is made of IECs that are often mediating signals between the two sides of the wall. Taking into consideration the pathogenesis of GvHD has been explained previously, this section is focused on APCs, T cells, and IECs.

### 3.1. Intestinal Epithelial Cells

Intestinal epithelial cells include absorptive epithelial, Paneth, and goblet cells. Their main role is to mediate signals between gut microbes and immune cells. Apart from that, they constitute a dense wall that eliminates the possibility of an influx of bacteria into the bloodstream. Additionally, these cells are responsible for the regulation of both the gut microbiota composition and immunomodulation [42].

During the conditioning before alloHCT, IECs are damaged, which results in the release of “danger signals”, such as TNFα or IL-1 [28]. Moreover, the destruction of these cells increases the permeability of the intestinal wall, which enables the translocation of luminal bacteria to the submucous tissue and the bloodstream. This in turn results in further release of “danger signals” and triggers the influx of immune cells [33].

### 3.2. Antigen-Presenting Cells

Antigen-presenting cells consist mainly of DCs and macrophages, and as discussed before, the role of host APCs is crucial in the pathogenesis of aGvHD [34]. The DCs occupying the intestinal wall (lamina propria) are an exceptional kind of DCs because of the presence of CD103 on their surface and the production of TGF-beta, which results in differentiation of naïve T cells into the T regulatory phenotype [43]. This seems to be extraordinary given the fact that other DCs are known producers of inflammatory cytokines and influence differentiation of T cells towards the Th1 phenotype. Moreover, the intestinal wall DCs present antigens in the mesenteric lymph nodes and usually do not reach the spleen, which could provoke commensal-specific systemic response [44].

Macrophages residing in the intestinal wall are exceptional as well. They do not express CD14, which causes LPS-induced cell activation and release of proinflammatory cytokines [45]. Macrophages share some features of DCs, such as the ability to affect differentiation of naïve T cells into the T regulatory phenotype. They can also tune the ability of intestinal DCs to drive the differentiation of Th17 cells [46]. Interestingly, the population of CD14^+^ macrophages that occupy the GI tract produces proinflammatory cytokines, such as TNFα and IL-23, which provokes the influx of similar macrophages to the intestinal wall [47].

To sum up, APCs are responsible for sensing the luminal antigens. Then, they are responsible for presenting the antigens to the T cells in the Peyer’s patches and mesenteric lymph nodes. The lack of balanced gut microbiota or the presence of some species of bacteria can provoke the production of proinflammatory cytokines and drive the differentiation of naïve T cells into the Th17 type [48]. Such a situation can influence the pathogenesis of aGvHD by additionally “boosting” the first step, which relies on the production of proinflammatory cytokines.

### 3.3. T Cells

The interplay between the gut microbiota and T cells could be limited to the balance between Tregs and Th17 cells. Many molecules derived from the lumen of the intestine can switch the mode of the intestinal immune system from proinflammatory to anti-inflammatory and vice versa through these two cell types. For instance, bacterial ATP stimulation can result in Th17 differentiation, but short-chain fatty acids (SCFAs) drive the differentiation of Tregs [49,50]. This is of great importance given the fact that Tregs are the main cells accountable for dampening the immune response, such as that seen in aGvHD [51]. Their tolerogenic capacity effectivity relies on the ability to downregulate the expression of TLR5, which is associated with aGvHD [52,53]. Another recently described regulatory cell type is the Th9 population that secretes IL-9, which was able to dampen IFNγ-mediated GvHD in the murine model [54].

## 4. The Role of Gut Microbiota in the Pathogenesis of Acute Graft-Versus-Host Disease

The first experiments assessing the role of the gut microbiota in the pathogenesis of aGvHD were performed in the 1970s, and they showed that mice treated with antibiotics targeting intestinal bacteria or germ-free animals developed less or a mild form of aGvHD [55,56]. Subsequent trials showed that introducing metronidazole and/or ciprofloxacin into the conditioning regimen (or other decontamination strategies) resulted in lower aGvHD incidence [57,58]. Apart from that, recent studies in the era of next-generation sequencing have shown that the loss of bacterial diversity is associated with the development of gastrointestinal aGvHD [59,60,61]. The lack of balance between Th17 and Treg cell differentiation due to the loss of diversified intestinal microbiota has been found to be the mechanism of such aGvHD induction [62]. However, it is interesting to note how the gut microbiota influences the mechanism of aGvHD pathogenesis.

The epithelial damage done by the conditioning therapy and irradiation leads to the loss of intestinal integrity followed by bacterial translocation. Moreover, the aGvHD itself contributes to further dysbiosis via damage to the Paneth cells, which facilitates the expansion of potentially harmful bacteria [63,64]. Bacteria carry pathogen-associated molecular patterns (PAMPs), which are accountable for the activation of the innate immune system [65]. PAMPs are recognized by several receptors, such as toll-like receptors (TLRs) [66], NOD-like receptors (NLRs) [67], and sialic acid-binding Ig-like lectins [68]. This leads to further activation of the donor T cells by the innate immune system and additionally exacerbates aGvHD.

Among the bacteria that prevent aGvHD development are Clostridiales, which are potent SCFAs producers, and their mechanism of action will be discussed further. The higher abundance of these bacteria was found to be associated with decreased number of GvHD-related deaths [69,70]. Jenq et al. showed that increased existence of bacteria from the *Blautia* genus belonging to Clostridiales was associated with decreased number of GvHD-related deaths. Additionally, the abundance of the same bacteria positively correlated with overall survival. The factors mentioned by the authors as possibly leading to the reduction of *Blautia* in the gut were treatment with antibiotics targeting the anaerobic bacteria and total parenteral nutrition administered for a long time [61]. Another study also showed that the use of aztreonam and cefepime, which both preserve anaerobic flora, resulted in a reduction in GvHD-related mortality [59].

On the other hand, the high abundance of some bacteria predisposes to GvHD onset and enhanced severity of the disease. *Enterococcus* is one such example, and increased number of these bacteria are found at the initiation of aGvHD [71]. Interestingly, the main source of energy for these bacteria is lactose, so patients with lactose malabsorption and subsequently increased lactose concentration in the intestinal lumen have an increased risk of *Enterococcus* domination. Moreover, an alloHCT mouse model on lactose-deprived diet showed lower number of these bacteria in the gut microbiota and thus improved survival related to GvHD [72]. Another bacteria highlighted to predispose to GvHD is *Akkermansia muciniphila*, which is associated with increased aGvHD lethality in mice [73].

Moreover, in addition to the bacteria themselves, their metabolites can also influence the pathogenesis of aGvHD. Probably the most known metabolite of intestinal bacteria is butyrate, one of SCFAs that is the main source of energy for IECs [74], and it is diminished after alloHCT [75]. Butyrate was shown to improve intestinal integrity, inhibit apoptosis, and in turn lead to alleviation of aGvHD [60]. Additionally, it causes increased differentiation of naïve intestinal T cells into Tregs, which are known for silencing the immune response associated with aGvHD [76,77]. Butyrate is also a histone deacetylase inhibitor that inhibits antigen-stimulated donor T cells [78]. Another metabolite produced by gut microbes is indole, which is a metabolite of tryptophan. Indole was shown to influence the expression of pro- and anti-inflammatory genes in IECs [71]. Patients with severe aGvHD had reduced 3-indoxyl sulfate levels and showed a shift toward *Enterococci* bacteria in the stool specimens [71]. Indoles administered orally in mice were found to limit aGvHD through the prevention and reparation of the lost mucosal barrier of the gut [79]. Moreover, a high concentration of choline-derived trimethylamine N-oxide (TMAO) was found to lead to increased lethality in the mouse model, which was attributed to induction of M1 macrophage polarization and subsequent differentiation of Th1 and Th17 cells [60]. Vitamin A also plays a role in the incidence of aGvHD, with studies showing that lower levels of vitamin A 30 days after alloHCT were associated with increased aGvHD prevalence in a pediatric population. This is probably because the differentiation of naïve T cells towards Tregs instead of Th17 is driven by vitamin A [80]. Moreover, commensal microbiota probably inhibits retinoid metabolism in enterocytes to prevent dysbiosis by decreasing the IL-22 levels. The notion that children with GI aGvHD had increased IL-22 supports this hypothesis [81,82].

Recently presented studies clearly suggest that the abundance and wealth of the gut microbiota during the neutrophil engraftment period is crucial regarding the particular time point when gut microbiota predicts the risk of aGvHD development. Researchers have shown that patients who have lowered bacterial diversity due to conditioning and antibiotics also have increased risk of aGvHD [83]. Another work by Golob et al. proved that the gut microbiota measured during the time of neutrophil recovery could become a useful predictor of subsequent aGvHD severity. The authors mentioned that such knowledge would allow physicians to act early and intensively to protect particularly prone patients based on their gut microbiota composition. In the same work, they showed that oral Actinobacteria and oral Firmicutes in the stool were positively correlated with severe aGvHD development, while some *Bacteroides*, such as *B. caccae*, *B. ovatus*, and *B. thetaiotaomicron*, were negatively correlated. Moreover, there could be an association between the gut microbiota of the HCT donor and recipient microbiota regarding the risk of aGvHD [84]. In line with this, Ingham et al. checked the dynamics of the gut, nose, and oral microbiota changes after alloHCT in a pediatric population and found that it decreased in all three sites directly after transplantation and reconstituted again 1–3 months after the procedure. Importantly, they proved that aGvHD could be predicted based on microbiota composition from all three sites before alloHCT. They also confirmed the effect of *Blautia* abundance on the risk and severity of aGvHD in the pediatric population [85].

## 5. Current Guidelines and Potential for the Future

The widely known first-line treatment for aGvHD is systemic steroid therapy [86], but the main limitation is relatively frequent refractoriness or dependency to steroids (35–50% of patients) [13]. Steroid-refractory aGvHD (SR-aGvHD) can be diagnosed after 3–5 days of treatment when we see clear progression or 5–7–10 days with no response [87]. When SR-aGvHD develops, the question is which therapy to use as a second-line treatment because there is still no consensus on this matter [88]. Moreover, the mortality in this group of patients is high, and the commonly applied treatment is ineffective. Therefore, the search for new drugs and new targets is critical. Some studies have shown an estimated two-year survival rate of 17% [13], and the average six-month survival estimate is 49% [14]. Additionally, what worsens the situation of these patients is a high frequency of infections, with bacterial infections (often with antibiotic-resistant bacteria) as the common etiology of deaths [89].

Among second-line therapies applied in SR-aGvHD, the best documented is ruxolitinib treatment [16]. Others therapies, such as extracorporeal photopheresis (ECP), mechanistic target of rapamycin kinase inhibitors, anti-TNFα antibodies, mycophenolate mofetil, anti-IL-2R antibodies, methotrexate, alemtuzumab, and antithymocyte globulin [14] are less effective or less studied [87]. In case of failure, it is recommended that another second-line treatment be tried rather than increasing the dose.

Several treatment options have already been evaluated in clinical studies. This includes ruxolitinib, as mentioned earlier, and Janus kinase (JAK) 1/JAK2 inhibitor, which influences all three steps of pathogenesis of aGvHD [90]. Another treatment that is emerging, with the possible status of being a “black horse”, is FMT (described in detail in the next paragraph), while other potential microbiota-derived products have also been proposed. Alpha1-antitrypsin (AAT) is also being studied in clinical trials. In murine models, AAT showed its efficacy by reducing the release of inflammatory cytokines and increasing the ratio of Treg to effector T cells. In phase 2 clinical trial in patients with SR-aGvHD (NCT01700036), the CR and ORR rates were 35 and 65%, respectively, after 28 days [91]. Anti-CD3/CD7 immunotoxin was given fast-track designation for SR-aGvHD treatment by the US Food and Drug Administration after a study found the CR and ORR rates were 50 and 60%, respectively, after 28 days. This therapeutic includes a mixture of anti-CD3 and anti-CD7 antibodies conjugated to recombinant ricin A, which suppresses T cell activation and causes their depletion in vivo [92]. Vedolizumab is another agent that has been studied in the treatment of aGvHD, but the results showed discrepancies [93,94,95]. It is a monoclonal antibody targeting integrin alpha4beta7 expressed on the surface of circulating lymphocytes and stops their relocation to the GI tract [96]. The mixed results can probably be attributed to the mechanism of action, which indicates that vedolizumab should only work in the early stages of the disease and not when aGvHD is already developed. Therefore, studies now concentrate on the role of vedolizumab as prophylaxis of GvHD (NCT03657160). Other therapies have also been studied but did not show positive results. Among them were agents such as brentuximab vedotin (anti-CD30 antibody–drug conjugate) [97] and begelomab (targeting CD26 expressed on the surface of CD4^+^ T cells) [98].

## 6. Fecal Microbiota Transplantation for the Treatment of Acute Graft-versus-Host Disease: What Do We Know Now?

Fecal microbiota transplantation is the therapy in which a healthy donor’s stool is transformed into fecal suspension and is given directly to the patient’s GI tract to reestablish balanced gut microbiota [25]. Given the key role of the gut microbiota in the pathogenesis of aGvHD, especially the intestinal form of aGvHD, it seems reasonable that FMT should have a positive influence on the gut barrier and limit step one in the three-step pathogenesis of aGvHD. Our review shows that in the published studies to date, the overall response rate of FMT in the treatment of gastrointestinal aGvHD could reach even 74%, with complete response accounting for 50% (see Table 1).

Kakihana et al. performed a pilot study of FMT in aGvHD of the gut on three patients with steroid refractoriness and one patient with steroid dependency. All patients responded to therapy, with three of them showing complete response and one showing a partial response. An increase in the number of effector regulatory T cells was also seen during response to FMT [99].

Spindelboeck et al. reported three patients with SR-GI-aGvHD who had a significantly dysbiotic gut microbiota. All of them responded to FMT, and an important finding was that microbial engraftment took place only after repeated FMT and was associated with reduced stool volumes [100].

A group of Chinese researchers performed a pilot study of FMT in SR-aGvHD on eight patients and showed great results, with all clinical symptoms being resolved and the gut microbiota composition being successfully restored. Compared to patients that did not receive FMT, those who received FMT showed significantly higher progression-free survival. Moreover, most importantly, no adverse events related to FMT were noted during and after infusion [101].

Shouval et al. presented a study of seven patients with GI-aGvHD, with six patients being steroid-refractory and one being steroid-dependent. Of note, this was the first study to be carried out with orally administered capsules for the treatment of GI-aGvHD, which proved to be well tolerated, safe, and efficient. The gut microbiota diversity after FMT increased, and 2 out of the 7 patients reaching complete response [25].

van Lier et al. published the results of a prospective, single-center, single-arm study of FMT treatment in 15 patients with steroid-refractory/dependent gastrointestinal aGvHD. FMT was well tolerated, and no adverse events were attributable to the procedure. In the study, 10 out of 15 patients reached complete remission within 1 month after FMT. Moreover, in 6 out of 10 responding patients, the immunosuppressive therapy could be successfully tapered. The alpha diversity of the gut microbiota increased, and the SCFAs-producing bacteria were enriched, including *Blautia* and Clostridiales species [103].

Kaito et al. reported a case of SR-aGvHD treated with FMT in the form of frozen capsules. In the study, FMT was delivered via a nasoduodenal tube. The composition of the gut microbiota was successfully restored, and symptoms, such as diarrhea, were resolved [104].

Zhong et al. presented two pediatric case reports, with one of them being an aGvHD case. Patients with aGvHD achieved complete remission of symptoms and had no infectious complications. The analysis of post-FMT stool microbiota showed reconstruction of diverse gut microbiota [105].

Biernat et al. presented two cases of aGvHD, but only one patient achieved complete remission of symptoms, with the second case unable to reach therapeutical efficacy. FMT was shown to eliminate the drug-resistant *Enterococcus* spp. but not the multidrug-resistant *Acinetobacter baumanii* or *Candida* spp. [106].

Mao et al. presented a case report of a SR-aGvHD patient who was treated with FMT in the form of oral capsules. After the first administration of FMT, the symptoms of aGvHD were relieved but recurred after 11 days. The second FMT resulted in durable remission of symptoms that were relieved during the two-month follow-up [107].

In a prospective, single-center study performed on 27 patients with GvHD (19 treated with FMT and 8 in the placebo group), Goloshchapov showed that eight patients reached complete response. Moreover, patients after FMT had higher overall “bacterial mass” and higher numbers of *E. coli*, *B. fragilis*, and *Bifidobacterium* spp. Of note, the “bacterial mass” in unresponsive patients was comparable to that seen in the placebo group [108]. The same authors conducted another study, this time on a pediatric population of seven patients aged 3–10 years with GI-aGvHD. In this study, complete response was reached in six patients by 120 days. Additionally, starting from day 8 after FMT, increased amounts of *B. fragilis*, *Faecalibacterium prausnitzii*, and *E. coli* were noted in the stool [109].

Goeser et al. presented a study on 11 patients with SR-GvHD and reported that nine of them achieved complete response. The stool frequencies and volume were significantly reduced. Additionally, the gut microbiota alpha diversity was increased and resembled the donor [110].

Zhao et al. enrolled 55 patients with SR-GI-GvHD, but only 41 of them with grade IV were included in the statistical analysis. A total of 23 patients were given FMT, while 18 were assigned to the placebo group. On day +90 after FMT, the FMT group showed significantly better OS. At the end of the study, the median survival time was 107 days for the control group and >539 days for the FMT group [111].

Our group performed a prospective study on acute gastrointestinal steroid-refractory/dependent GvHD treatment with FMT. A total of 16 FMTs were performed in 11 patients with aGvHD and 2 patients with chronic GvHD. Complete response (CR) was reached in 42%, while the overall response rate (ORR) was 57%. Moreover, we analyzed the decolonization status of patients colonized with antibiotic-resistant bacteria before FMT and showed that 71% of patients were at least partially decolonized [23]. Recently, we presented a case study of four patients treated with FMT in combination with ruxolitinib and showed great effectiveness (3 of 4 patients achieved CR). Interestingly, the results were better and faster when we diagnosed patients with steroid refractoriness/dependency earlier and immediately introduced second-line therapy (ruxolitinib with FMT sessions), which indicates that FMT works better when the intestinal barrier is still in a good condition [21]. 

Malard et al. recently presented the results from a Phase IIa HERACLES Study and Expanded Access Program (EAP) [24]. A total of 76 patients with SR-GI-aGvHD were treated with pooled-donor, high-richness microbiota therapeutic MaaT03, and 29 of them reached complete response. The ORR on day 28 post FMT was 38 and 60% for the HERACLES and EAP groups, respectively. Furthermore, the OS was significantly higher [24].

There are also a few ongoing trials studying the effectiveness of FMT in the treatment of aGvHD (NCT04269850, NCT03819803, NCT03812705, NCT04285424, and NCT03359980).

## 7. Safety of FMT

An analysis of FMT procedures conducted in the last two decades showed an adverse events (AEs) rate of 19% [112]. It is worth mentioning that most of them were mild and self-limiting, and only 1.39% of procedures were complicated by severe adverse events. The most common AEs were diarrhea, abdominal pain, nausea, and vomiting. The rate of adverse events depended on the manner of FMT administration and its indication. For instance, FMT conducted via the upper gastrointestinal tract was associated with more adverse events than methods of microbiota transplantation via the lower gastrointestinal tract. The highest rate of adverse events was reported when FMT was given for infections with antibiotic-resistant bacteria. During the last two decades, only four cases of deaths were associated with FMT (one case was assessed as probably associated) [112], and we calculated the risk of death associated with microbiota transplantation as 0.02%.

The preparation of washed FMT was recently proposed and showed significantly increased safety compared to manual microbiota transplantation [113,114].

## 8. Conclusions

To sum up, there are big expectations for FMT in treatment of aGvHD, which has been prompted by its encouraging effectiveness so far, with very few serious adverse events (common even without FMT in this group of patients). Therefore, if confirmed by randomized clinical trials, we believe that the use of FMT could be the standard in the treatment of steroid-refractory or -dependent aGvHD. Furthermore, we can imagine that FMT may not be just limited to patients with steroid refractoriness or dependency in the future and rather applied more broadly to patients with aGvHD as a first-line therapy (sparing the adverse events associated with steroid therapy) or prophylaxis. Moreover, as every patient undergoing alloHCT would have performed a gut microbiota profiling, FMT may be used to re-establish the gut microbiota and increase integrity of the gut barrier. This alone could be enough prophylaxis against acute and chronic GvHD. Of course, more evidence with randomized clinical trials are needed.

Great hopes are also associated with targeted therapies derived from the gut microbiota, such as live biotherapeutic products.

We believe that restoring natural barriers and compositions destroyed by the severity of treatment itself and re-establishing them to their original state, or even to a “perfect” state, will be widely discussed in the future.

## Figures and Tables

**Table 1:** Clinical studies and trials on fecal microbiota transplantation in GvHD treatment.

Study	Indication/Population	Number of Patients	Administration Route	Study Type	Donor Relation	Total Number of FMTs	Serious Adverse Events	Response
Kakihana et al. [99]	Steroid-resistant/dependent gut GvHD	4	Nasogastric tube	Prospective	Spouse/relative	7	1 lower GI bleeding, hypoxemia (probably not related)	*n =* 3, CR; *n =* 1, PR
Spindelboeck et al. [100]	Steroid-resistant grade IV gut GvHD	3	Colonoscopy	Retrospective, case series	Unrelated/sibling	9	No serious AEs	*n =* 2, CR; *n =* 1, PR
Qi et al. [101]	Steroid-resistant GvHD	8	Nasoduodenal tube	Prospective	Unrelated	12	No serious AEs	*n =* 5, CR; *n =* 1, PR
Shouval et al. [102]	Steroid-resistant/dependent GvHD	7	Oral capsules	Prospective	Unrelated	15	2 bacteremia (deemed unrelated)	*n =* 2, CR
van Lier et al. [103]	Steroid-resistant/dependent GvHD	15	Nasoduodenal tube	Prospective	Unrelated	15	No serious AEs	*n =* 11, CR
Kaito et al. [104]	Steroid-resistant grade IV gut GvHD	1	Oral capsules	Prospective	Unrelated	2	No serious AEs	*n =* 1, PR
Zhong et al. [105]	Steroid-resistant grade III gut GvHD	1	Nasoduodenal tube	Retrospective	Unrelated	2	No serious AEs	*n =* 1, CR
Biernat et al. [106]	Steroid-resistant grade IV gut GvHD	2	Nasoduodenal tube	Retrospective	Unrelated	7	No serious AEs	*n =* 1, CR
Mao et al. [107]	Steroid-resistant grade IV gut GvHD	1	Oral capsules	Retrospective, case report	Unrelated	2	No serious AEs	*n =* 1, CR
Goloshchapov et al. [108]	Steroid-resistant GvHD/4-overlap GvHD	19	3 gastroscopy, 3 nasointestinal tube, 13 oral capsules	Prospective	15 unrelated, 4 related	19	No data	*n =* 8, CR; *n =* 8, PR
Goloshchapov et al. [109]	Steroid-resistant GvHD/2-overlap GvHD	7	2 gastroscopy, 2 nasoduodenal tube, 3 oral capsules	Prospective pediatric	4 unrelated, 3 related, All were also HSC donors	7	No serious AEs	*n =* 5, CR; *n =* 1, PR
Goeser et al. [110]	Steroid-resistant GvHD	11	9 oral capsules, 2 nasojejunal tube	Retrospective, case series	Unrelated	11	No serious AEs	*n =* 9, CR; *n =* 2, PR
Zhao et al. [111]	Steroid-resistant GvHD	23	Nasoduodenal/nasogastric tube	Prospective	Unrelated	43	2 thrombocytopenia and cardiac events	*n =* 13, CR; *n =* 3, PR
Biliński et al. [23]	Steroid-resistant GvHD	11	Nasoduodenal tube	Prospective	Unrelated	14	2 sepsis and septic shock	*n =* 5, CR; *n =* 1, PR
Biliński et al. [21]	Steroid-resistant GvHD	4	Nasoduodenal tube	Prospective	Unrelated	15	No serious AEs	*n =* 3, CR
Malard et al. [24]	Steroid-resistant grade III–IV gut aGvHD *n =* 24, Steroid-dependent or Steroid-resistant gut aGvHD (classical *n =* 41, late onset *n =* 3, overlap syndrome *n =* 8) for Expanded Access Program	76	2 nasogastric tube, 74 enema	Prospective	Pooled unrelated	192	5 serious AEs in 2 patients	*n* =29, CR; *n* = 14, VGPR *n* = 5, PR
**TOTAL**		**193**				**372**	**12 (4.8%)**	**ORR (CR + VGPR + PR) = 74% CR = 50%**

Abbreviations: aGvHD, graft-versus-host disease; FMT, fecal microbiota transplantation; AE, adverse event; CR, complete remission; PR, partial remission; ORR, overall response rate; GI, gastrointestinal.
